# Supplementary material for: Deletion of wfs1 Impairs Oligodendrocyte Precursor Cells Dorsal Distribution and Myelination Through the wfs1-hmgcs1 Axis in Zebrafish
Source: Biology (Basel). 2026 Aug 16;15(16):1402. doi: 10.3390/biology15161402 (PMC13510151; doi:10.3390/biology15161402)
Supplement: Supplementary file 1 [file biology-15-01402-s001.zip › File S1-Transcriptome sequencing data and differentially expressed genes (DEGs) in wfs1-- zebrafish at 5 dpf/2.KEGG/wfs1KOvsWT/ALL/src/dre04814.html]

dre04814


- dre:406297

- Up regulated genes

101882034(7.07443480885884)

- dre:556023

- Up regulated genes

556023\_1(2.75083665747002)

- Down regulated genes

556023(-1.34487366325401)
- dre:799461

- Up regulated genes

799461\_2(3.03083243104682)
- dre:100334351

- Up regulated genes

100334351(1.28589859912917)

- dre:474327

- Up regulated genes

474327(1.18169964792452)

- Down regulated genes

474327\_1(-5.01402477912525)

- dre:431777

- Up regulated genes

431777(1.35055689210441)

- dre:336681

- Down regulated genes

406811\_1(-1.09337424046752)

- dre:562052

- Up regulated genes

562052\_1(1.00437130309591)

- dre:571487

- Up regulated genes

571487(1.74295403341254)

- dre:334274

- Down regulated genes

334274(-1.53299620220276)
- dre:100149148

- Down regulated genes

100149148(-1.048436973731)

- dre:100037358

- Down regulated genes

100037358\_1(-1.14225112918381)

- dre:337226

- Up regulated genes

337226\_1(4.74603512488049)

- Down regulated genes

337226(-2.98693262894109)
- dre:798632

- Up regulated genes

798632(1.33935841885554)

- dre:677750

- Up regulated genes

677750(1.37913343503877)

- dre:30429

- Up regulated genes

30429(3.98975094921512)

- Down regulated genes

30429\_2(-1.78714384880949)

- dre:57934

- Up regulated genes

57934\_1(9.91304974129429)

- Down regulated genes

57934(-1.25539807424297)

- dre:353247

- Down regulated genes

353247(-1.16019504230133)

- dre:353247

- Down regulated genes

353247(-1.16019504230133)

Close
